# Supplementary material for: A Critical View of the Application of the APEX Software (Aqueous Photochemistry of Environmentally-Occurring Xenobiotics) to Predict Photoreaction Kinetics in Surface Freshwaters
Source: Molecules. 2019 Dec 18;25(1):9. doi: 10.3390/molecules25010009 (PMC7017383; doi:10.3390/molecules25010009)
Supplement: Supplementary file 1 [file molecules-25-00009-s001.zip › 3.2.4_gcc-4.4.0.zip!/3.2.4_gcc-4.4.0/3.2.4_gcc-4.4.0/doc/octave/html/interpreter/Indexing-Objects.html]

Indexing Objects - Untitled


Next: Overloading Objects,
Previous: Manipulating Classes,
Up: Object Oriented Programming


---

### 33.3 Indexing Objects

Objects can be indexed with parentheses, either like
a `(`idx`)` or like a `{`idx`}`, or even
like a `(`idx`).`field. However, it is up to the user
to decide what this indexing actually means. In the case of our polynomial
class p `(`n`)` might mean either the coefficient of the
n-th power of the polynomial, or it might be the evaluation of the
polynomial at n. The meaning of this subscripted referencing is
determined by the `subsref` method.

— Built-in Function: **subsref** (val, idx)  
> Perform the subscripted element selection operation according to
> the subscript specified by idx.
>
> The subscript idx is expected to be a structure array with
> fields type and subs. Valid values for type
> are "()", "{}", and ".".
> The subs field may be either ":" or a cell array
> of index values.
>
> The following example shows how to extract the two first columns of
> a matrix
>
> ```
>           val = magic(3)
>                => val = [ 8   1   6
>                           3   5   7
>                           4   9   2 ]
>           idx.type = "()";
>           idx.subs = {":", 1:2};
>           subsref(val, idx)
>                => [ 8   1
>                     3   5
>                     4   9 ]
> ```
>
> Note that this is the same as writing `val(:,1:2)`.
>
>
>
> **See also:** subsasgn, substruct.

For example we might decide that indexing with "()" evaluates the
polynomial and indexing with "{}" returns the n-th coefficient (of n-th power).
In this case the `subsref` method of our polynomial class might look like

```
```
     function b = subsref (a, s)
       if (isempty (s))
         error ("polynomial: missing index");
       endif
       switch (s(1).type)
         case "()"
           ind = s(1).subs;
           if (numel (ind) != 1)
             error ("polynomial: need exactly one index");
           else
             b = polyval (fliplr (a.poly), ind{1});
           endif
         case "{}"
           ind = s(1).subs;
           if (numel (ind) != 1)
             error ("polynomial: need exactly one index");
           else
             if (isnumeric (ind{1}))
               b = a.poly(ind{1}+1);
             else
               b = a.poly(ind{1});
             endif
           endif
         case "."
           fld = s.subs;
           if (strcmp (fld, "poly"))
             b = a.poly;
           else
             error ("@polynomial/subsref: invalid property \"%s\"", fld);
           endif
         otherwise
           error ("invalid subscript type");
       endswitch
       if (numel (s) > 1)
         b = subsref (b, s(2:end));
       endif
     endfunction
```
```

The equivalent functionality for subscripted assignments uses the
`subsasgn` method.

— Built-in Function: **subsasgn** (val, idx, rhs)  
> Perform the subscripted assignment operation according to
> the subscript specified by idx.
>
> The subscript idx is expected to be a structure array with
> fields type and subs. Valid values for type
> are "()", "{}", and ".".
> The subs field may be either ":" or a cell array
> of index values.
>
> The following example shows how to set the two first columns of a
> 3-by-3 matrix to zero.
>
> ```
>           val = magic(3);
>           idx.type = "()";
>           idx.subs = {":", 1:2};
>           subsasgn (val, idx, 0)
>                => [ 0   0   6
>                     0   0   7
>                     0   0   2 ]
> ```
>
> Note that this is the same as writing `val(:,1:2) = 0`.
>
>
>
> **See also:** subsref, substruct.

Note that the `subsref` and `subsasgn` methods always receive the
whole index chain, while they usually handle only the first element. It is the
responsibility of these methods to handle the rest of the chain (if needed),
usually by forwarding it again to `subsref` or `subsasgn`.

If you wish to use the `end` keyword in subscripted expressions
of an object, then the user needs to define the `end` method for
the class.

For example the `end` method for our polynomial class might look like

```
```
     function r = end (obj, index_pos, num_indices)
     
       if (num_indices != 1)
         error ("polynomial object may only have one index")
       endif
       
       r = length (obj.poly) - 1;
     
     endfunction
```
```

which is a fairly generic `end` method that has a behavior similar to
the `end` keyword for Octave Array classes. It can then be used for
example like

```
     p = polynomial([1,2,3,4]);
     p(end-1)
     => 3
```

Objects can also be used as the index in a subscripted expression themselves
and this is controlled with the `subsindex` function.

— Function File: idx = **subsindex** (a)  
> Convert an object to an index vector. When a is a class object
> defined with a class constructor, then `subsindex` is the
> overloading method that allows the conversion of this class object to
> a valid indexing vector. It is important to note that
> `subsindex` must return a zero-based real integer vector of the
> class "double". For example, if the class constructor
>
> ```
>           function b = myclass (a)
>            b = myclass (struct ("a", a), "myclass");
>           endfunction
> ```
>
> then the `subsindex` function
>
> ```
>           function idx = subsindex (a)
>            idx = double (a.a) - 1.0;
>           endfunction
> ```
>
> can then be used as follows
>
> ```
>           a = myclass (1:4);
>           b = 1:10;
>           b(a)
>           => 1  2  3  4
> ```
>
>
>
>
> **See also:** class, subsref, subsasgn.

Finally, objects can equally be used like ranges, using the `colon`
method

— Function File: r = **colon** (a, b)  
— Function File: r = **colon** (a, b, c)  
> Method of a class to construct a range with the `:` operator. For
> example.
>
> ```
>           a = myclass (...)
>           b = myclass (...)
>           c = a : b
> ```
>
>
>
>
> **See also:** class, subsref, subsasgn.
